# Long-term mortality after stroke is higher than after myocardial infarction

**DOI:** 10.1007/s10072-016-2502-4

**Published:** 2016-02-10

**Authors:** K. Chwojnicki, Ł. Wierucki, P. Zagożdżon, B. Wojtyniak, W. M. Nyka, T. Zdrojewski

**Affiliations:** Department of Neurology, Medical University of Gdańsk, Dębinki 7 Street, 80-211 Gdańsk, Poland; Department of Arterial Hypertension and Diabetology, Medical University of Gdańsk, Dębinki 7 Street, 80-211 Gdańsk, Poland; Department of Hygiene and Epidemiology, Medical University of Gdańsk, Dębinki 7 Street, 80-211 Gdańsk, Poland; Polish Institute of Public Health, Chocimska 24 Street, 00-230 Warsaw, Poland

**Keywords:** Ischemic stroke, Myocardial infarction, Long-term mortality

## Abstract

Mortality caused by coronary heart disease and ischemic stroke (IS) in Poland is still among the highest in Europe. Because acute myocardial infarction (AMI) and IS share major common risk factors, it would be expected that trends in long-term mortality (LTM) and incidence of these two diseases would be similar. Nevertheless, better AMI acute phase therapy and older age of IS patients make post-IS and post-AMI prognosis difficult to compare. The aim of the study was to verify the thesis that, regardless of age and sex, the long-term prognosis is worse for post-IS than for post-AMI subjects. The study was conducted in Polish city—Gdynia (250,000 of inhabitants) among 997 subjects (464 post-IS, 533 post-AMI) randomly selected from all post-IS and post-AMI patients, witch survived hospitalization period in years 2000–2005. The observation period varied from 1 month to 11 years. LTM was shown as standardized mortality ratios. Kaplan–Meyer survival curves and Cox proportional hazard regression model were used to compare LTM in post-IS and post-AMI subjects. Post-IS and post-AMI groups did not differ by sex or age of event. Fewer deaths were recorded in post-AMI group (38.8 vs. 51.5 %, OR 0.60, 95 % CI 0.46–0.77). This difference was most evident in males (39.7 vs. 57.8 %, OR 0.48, 95 % CI 0.34–0.66). Kaplan–Meyer estimates showed faster reduction of survival probability in the post-IS males. In Cox regression model presence of IS increased long-term mortality in males. Long-term prognosis was worse for post-IS males in comparison with post-AMI population from Gdynia.

## Introduction

Coronary heart disease (CHD) and stroke are leading causes of death and disability [1]. In Poland and in other Central and Eastern European countries the morbidity and mortality rates are among the highest in Europe [1]. Corresponding rates in USA and Western Europe have been decreasing over the past 30 years, mainly as the result of new drugs, better control of cardiovascular risk factors and implementation of revascularization procedures [3–5].

Since 1991 mortality caused by CHD has also been decreasing in Poland. [1] An analysis using the epidemiological model IMPACT carried out in 2012 showed that this decline is due to lifestyle changes and development of new treatment strategies—drugs and revascularization procedures [6].

In the review of Feigin et al. [7] (data from Poland not included) there is a statistically significant trend in stroke incidence rates over the past four decades, with a 42 % decrease in high-income countries and a >100 % increase in low- to middle-income countries.

It seems possible that we will witness a steady increase in the incidence of stroke in poorer countries due to the unfavorable demographic trend. Forecasts predict a tripling of the population aged >80 years in Europe to 2060, and the risk of stroke in the 80-year-old patient is 100× greater than in 40-year-old one. Equally important is the age-dependent increase in the risk of CHD [8–10].

Despite the decline in mortality from CHD, overall cardiovascular mortality remains high because of death rates from ischemic stroke [11].

Because CHD and ischemic stroke (IS) share major common risk factors, it would be expected that trends in long term mortality and incidence of these two major cardiovascular diseases would be similar. Nevertheless, studies have found that changes and levels of rates of IS and CHD may not show similar patterns in a population [2].

Acute myocardial infarction (AMI), which is the main complication of CHD, in the short term has a better prognosis than IS. This is determined by different factors: medical (more efficient methods for the acute phase treatment), organizational and financial (more professional cardiac wards) and social (IS patients are 5–10 years older with greater comorbidity). However, in recently published studies of 1-year mortality after AMI it is found to be surprisingly high and ranges from 10 to 20 % [12–14].

Studies assessing the long-term prognosis after AMI and IS usually present data for AMI and IS separately. Few studies directly comparing mortality from IS and AMI are heterogeneous and age limited [15].

## Aim

The aim of the study was to verify the thesis that, regardless of age and sex, the long-term prognosis is worse for IS than AMI.

## Methodology

The study was observational. It was conducted among the residents of Gdynia (a big city in the north of Poland, with 250,000 inhabitants). Patients suffering from AMI or IS in 2000–2005 were identified based on hospital records. The IS respondents were selected according to the definition of IS created by WHO in 1990 (“focal or global disturbance of cerebral function, with symptoms lasting 24 h or longer, with no apparent cause other than of vascular origin”) [16]. In the case of the AMI group the definition used was that of the European Society of Cardiology and the American College of Cardiology from 2000 (based on troponin/CK-MB levels and ECG ischemic changes/coronary intervention) [17].

Of all the identified patients (3410 IS and 5502 AMI), hospitalized in 2000–2005 and who survived the hospitalization period, 500 post-IS and 550 post-AMI subjects were randomly selected. Stratified sampling method was applied. The selection was conducted in proportion to sex and age group (31–50, 51–60, 61–70 and 71–80 years). The sample size was calculated to detect a difference in mortality between the IS and AMI groups at the level of 5 % at a power test of 0.9 for the entire period of the follow-up. The missing data was assumed at the level of 10 %.

The deaths and their causes were identified by the end of 2011 according to the database of the Polish Institute of Public Health, which is a government agency responsible for monitoring the health status of the population.

All received from hospitals and Polish Institute of Public Health records were anonymous and deprived of identifying information. For this reason informed consent should not be obtained.

### Statistical analysis

The IS and AMI subjects were compared in terms of overall and annual mortality. Continuous variables at time of follow-up and age were expressed as mean and standard deviation (SD) or median. Categorical variables (sex, AMI or IS group, deaths) were expressed as percentage. Simple associations were performed using *T* test or *U* Mann–Whitney test for continuous variables and Chi square test for categorical variables. *p* values were two-tailed and a value <0.05 was considered to be statistically significant. For Kaplan-Meyer survival curves comparison, a log rank test was performed. Multivariate analyses were performed with proportional hazards regression model (Cox model, Breslow method for ties) adjusted by age, sex, their interaction and type of event. Standardized mortality ratios (SMRs) and standardized death rates (SDRs) for IS and AMI groups were calculated using the data on deaths and age structure of the general population of Tricity (Gdańsk, Gdynia, Sopot). SMRs were calculated with the indirect method of standardization. Average values for 2006 for the Tricity mortality rates for each age group and sex combination were used as the reference rates (for the latter the data was published by Central Statistical Office of Poland).

## Results

Of the 1050 patients, randomly selected and meeting the inclusion criteria, data on mortality and causes of death were obtained from 997 (464 IS, 533 AMI). The IS and AMI groups did not differ by sex or age of onset. Median of follow-up period in both groups was very similar (Table 1). Fewer deaths were recorded in the AMI group (38.8 % AMI vs. 51.5 % IS, OR 0.60, 95 % CI 0.46–0.77). This difference was most evident when comparing men in both groups (39.7 % AMI, 57.8 % IS, OR 0.48, 95 % CI 0.34–0.66, *p* < 0.001). The proportion of deaths among women with AMI and IS did not differ significantly (37.2 % AMI, 43.8 % IS; OR 0.76, 95 % CI 0.5–1.15; *p* = 0.19).

**Table 1:** Baseline characteristics of IS and AMI populations

	Post-IS group		Post-AMI group	
	*N*	%	*N*	%
Patients selected to the study	500	100.0	550	100.0
Patients followed in the study	464	100.0	533	100.0
Sex				
Women	208*	44.8	180*	33.8
Men*	256*	55.2	353*	66.2
Deaths from 2000 to 2011*				
Women				
30–64	27*	29.3	8*	12.5
>64	64*	55.2	59	50.9
>30	91*	43.8	67	37.2
Men				
30–64*	59*	46.1	51*	27.6
>64	89*	69.0	89	53.0
>30	148*	57.8	140	39.7
All**	239	51.5	207	38.8
Age at event				
Mean ± SD	63.3 ± 9.6		62.3 ± 9.6	
Median ± IR	65 ± 15		65 ± 15	
Years of follow-up (median)	8.5		8.0	
Age of death (mean ± SD)	74.0 ± 7.6		72.0 ± 8.8	

*SD* standard deviation, *IR* interquartile range

* *p* < 0.05 for men–women comparisons (Chi square test)

** *p* < 0.05 for IS–AMI comparison (Chi square test)

**Fig. 1:**
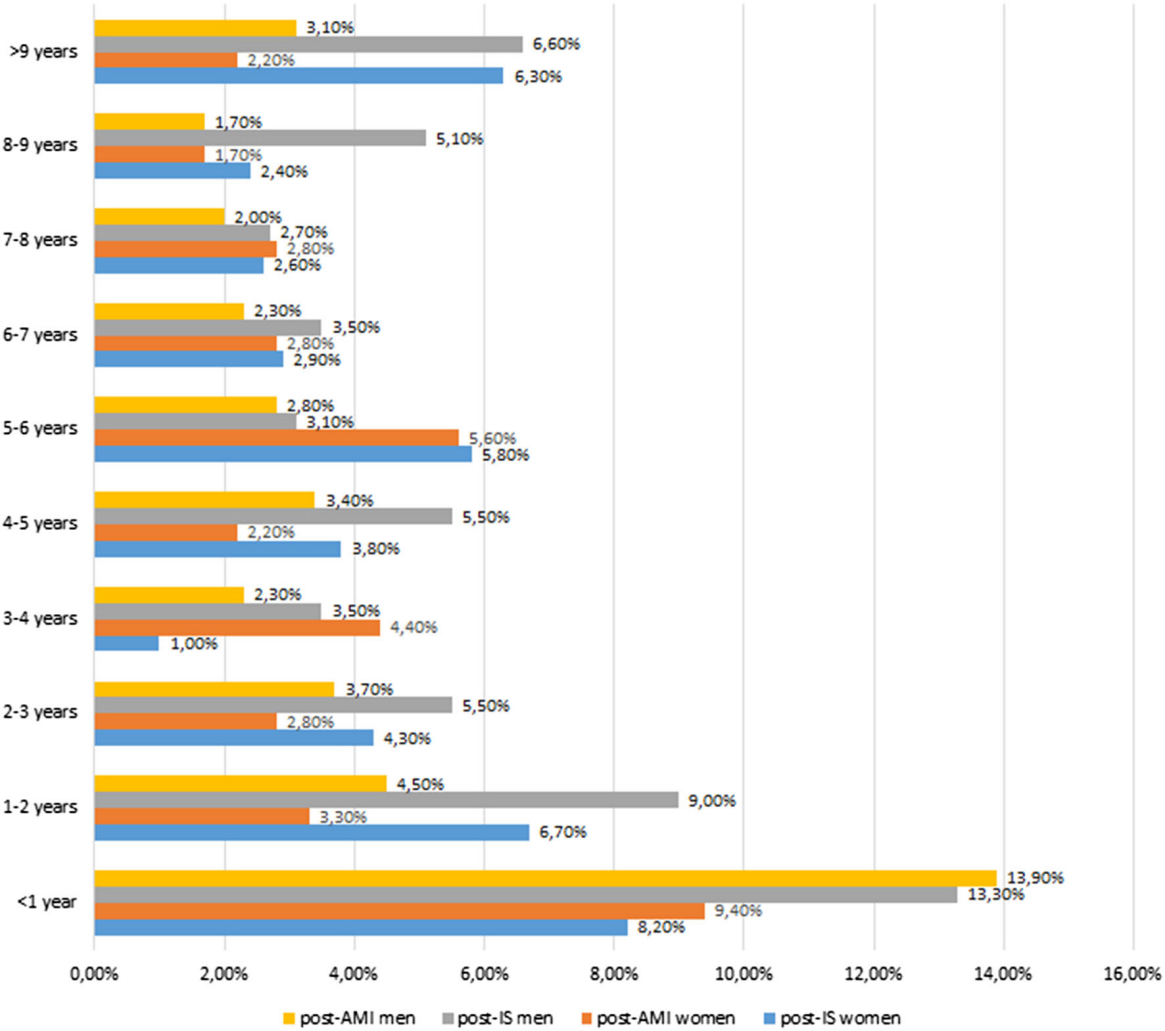
Proportion of deaths in IS and MI group

In both groups, the proportion of deaths was highest during the first year since a cardiovascular event (Fig. 1). Then this ratio showed a significant decrease in the AMI group, while a similar decline occurred in the IS group only after 2 years since the event.

Similar differences were found when comparing the SDR in the IS and AMI groups. SDR for the entire follow-up period was considerably higher in the whole IS group (IS 77.04, 95 % CI 67.71–87.64, AMI 58.14, 95 % CI 50.61–66.78). This relation occurred for both sexes, in the case of men, however, it was stronger (Table 2).

**Table 2:** Crude death rates and 95 % CI in IS and AMI groups according to sex and years since event

SDR, 95 % CI			*p*
Years from event	Post-IS group	Post-AMI group	
Women			
(0–1]	89.85; 55.86–144.53	103.22; 64.17–166.04	0.034
(1–2]	80.04; 47.40–135.14	39.08; 17.56–86.99	<0.001
(2–4]	34.41; 19.06–62.14	45.61; 26.48–78.54	0.047
(4–6]	64.06; 40.86–100.44	45.90; 26.07–80.83	0.008
(6–8]	41.48; 22.97–74.91	46.74; 25.15–86.86	0.112
>8	84.19; 52.34–135.43	66.41; 31.66–139.31	0.014
Total	61.49; 49.95–75.69	54.90; 43.05–70.00	0.088
Men			
(0–1]	147.40; 104.79–207.34	153.99; 116.38–203.75	0.107
(1–2]	114.76; 76.26–172.70	56.83; 34.82–92.77	<0.001
(2–4]	62.66; 41.26–95.17	39.70; 25.88–60.88	<0.007
(4–6]	71.58; 47.13–108.71	45.80; 30.16–69.56	<0.004
(6–8]	54.91; 33.10–91.08	34.14; 20.22–57.66	0.009
>8	138.23; 94.79–201.56	54.82; 31.83–94.41	<0.001
Total	91.54; 77.66–107.91	59.84; 50.55–70.83	<0.001
All			
(0–1]	121.04; 91.74–159.70	136.67; 107.38–173.96	0.031
(1–2]	98.58; 71.42–136.06	50.57; 33.30–76.80	<0.001
(2–4]	49.20; 34.98–69.21	41.77; 29.84–58.45	0.089
(4–6]	67.89; 49.99–92.20	45.84; 32.75–64.15	0.008
(6–8]	48.30; 32.88–70.93	38.46; 25.78–57.39	0.051
>8	110.76; 82.43–148.84	58.39; 37.67–90.50	<0.001
Total	77.04; 67.72–87.64	58.14; 50.61–66.78	0.010

*SDR* standardized death rate

In both groups the highest values of SDR were noticed during the first year of follow-up. In the IS group the SDR was also high during the second year since event (in the AMI group it dropped significantly after 1 year). SDR increased again after 8 years of follow-up in the two groups, which was probably due to the aging of the populations (Table 2). SDR were generally higher for the population of men, clearly within the IS group, slightly in the case of AMI. SDR values observed during the follow-up point to a continually increased risk of death compared to the reference population (population of Gdansk aged 30–80 years old, where the death rate is 9.43/1000 (for women—6.64, for men—12.27). The highest values of SDR were noted for younger patients—in the group of 30–45 year olds they were even 150 times greater than in the reference population (in the first year of follow-up every fifth patient died after a stroke and every sixth after AMI).

SMR for the entire period of observation was 4.68 (CI 4.11–5.32) for IS and 3.51 (CI 3.06–4.03) for AMI. Quotients of SMR IS/SMR AMI hazard ratio according to sex and years since event are shown in Table 3. Quotient is the highest in the first year and also after sixth year of follow-up. Moreover, its value is higher among men.

**Table 3:** Quotient of SMR IS/SMR AMI hazard ratio according to sex and years since event

Years from event	Women	Men	All
(0–1]	1.412	1.673	1.545
(1–2]	0.974	0.715	0.805
(2–4]	1.273	1.255	1.282
(4–6]	0.772	1.347	1.142
(6–8]	4.226	3.581	5.638
>8*	2.762	1.194	2.088
Total	1.190	1.506	1.332

* *p* < 0.05 for women vs men comparison

**Fig. 2:**
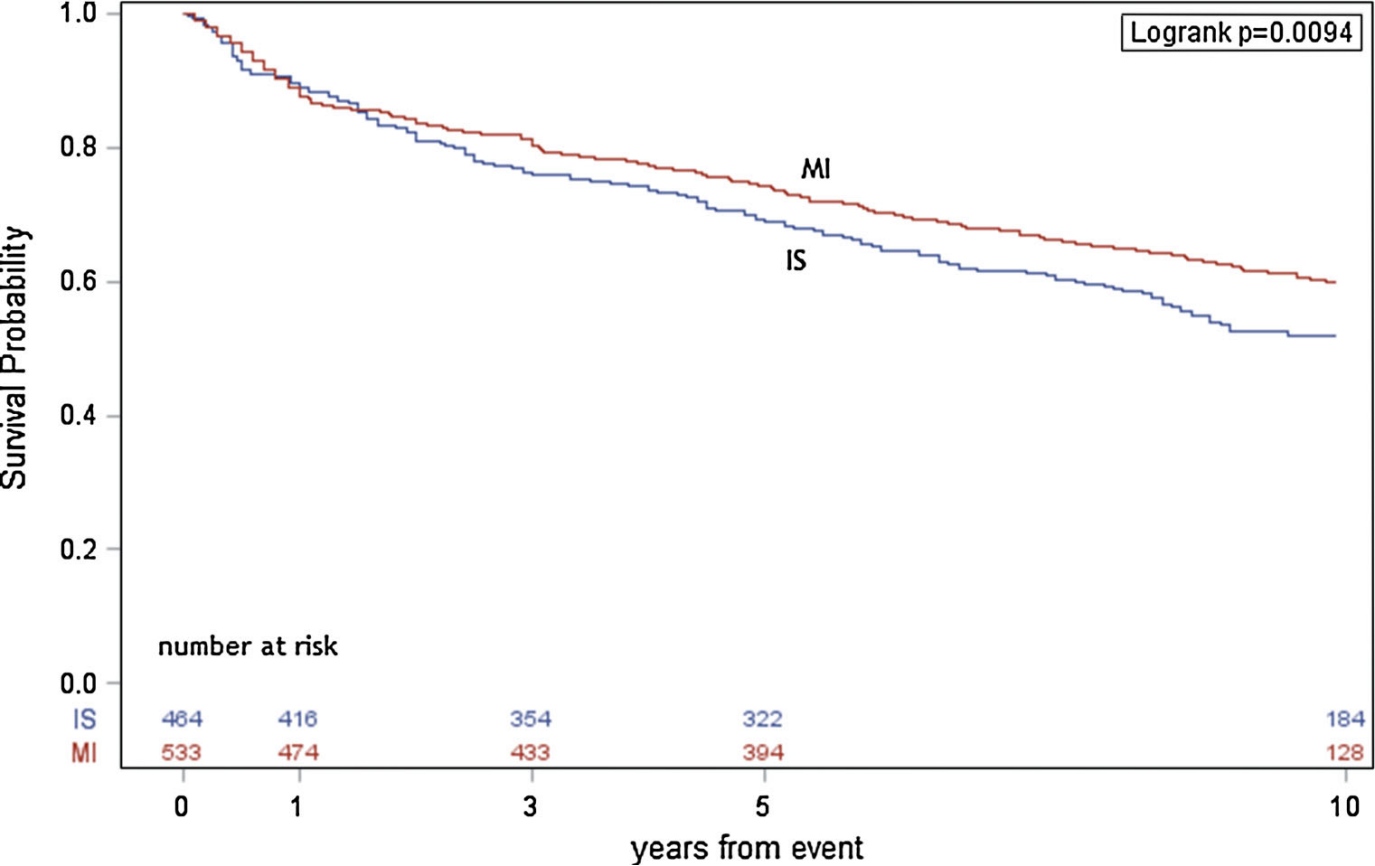
Kaplan–Meyer survival curves for AMI and IS groups

**Fig. 3:**
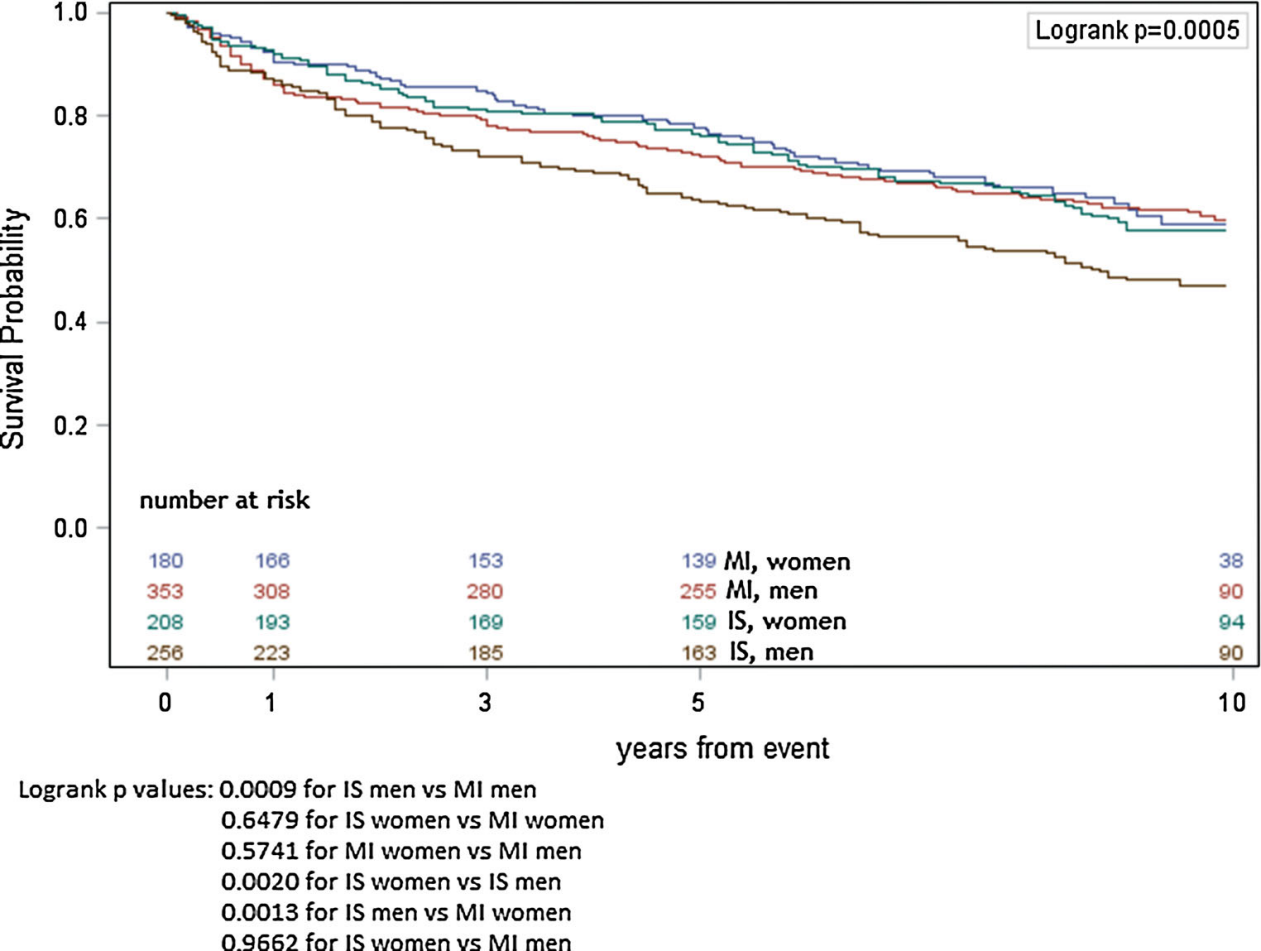
Kaplan–Meyer survival curves for sex specific AMI and IS groups

Kaplan–Meyer estimates of survival probability with the number at risk for IS and AMI subjects respectively also show faster reduction of survival probability (SP) in the IS group (Fig. 2). Kaplan–Meyer estimates calculated separately for women and men after IS or AMI present the lowest survival probability for men after IS, SP for women after IS, women after AMI and men after AMI are similar (Fig. 3).

A predictive model of death (Cox proportional hazards regression model), including type of event, sex, their interaction and age (every 5 years), confirmed that presence of stroke declined long-term survival in males. Older age was also independent predictor of death (Table 4).

**Table 4:** A predictive model of death (Cox proportional hazards regression model)

Parameter	Pr. > Chi square	Hazard ratio	95 % CI for hazard ratio	
Model without event × sex interaction				
Type of event (stroke)	0.0041	1.317	1.091	1.589
Sex (male)	0.0014	1.378	1.133	1.677
Age (every 5 years)	<0.0001	1.252	1.185	1.322
Model with event × sex interaction				
Type of event (stroke)	0.5578	–	–	–
Sex (men)	0.2675	–	–	–
Age (every 5 years)	<0.0001	1.252	1.185	1.322
Event × sex				
Stroke, female	0.0168	1.199	0.901	1.508
Stroke, male		1.448	1.249	1.724

**Fig. 4:**
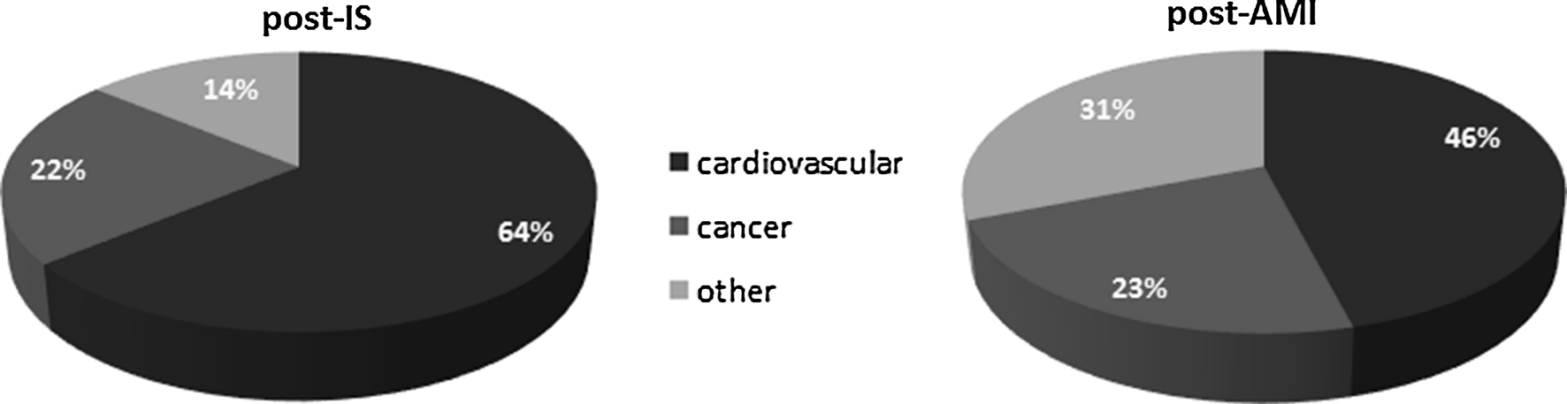
Causes of death among post-IS and post-AMI subjects

In terms of causes of death, the AMI group did not differ from the general population in Poland. In the IS group, there were more deaths from vascular causes. The number of deaths due to cancer was similar in both groups (Fig. 4).

## Discussion

There are numerous publications on early and long-term prognosis after cardiovascular events. However, they do not include direct comparisons in terms of mortality after IS and AMI. On the one hand, these studies show a high long-term mortality after both AMI and IS; on the other hand, they point to an improvement in early prognosis over the past 30 years.

The AMI mortality improvement is attributable to specific cardiologic treatments and changes in risk factors, for IS it is also a result of organization of stroke units.

Yet, the positive trend of declining mortality is much stronger after AMI, and the improvement of post-stroke prognosis is relatively small even in Western European countries such as Denmark or Finland, which have a very good organization of stroke care [15]. It is worth mentioning that thrombolytic therapy known as the most effective in treating the acute phase of IS does not affect mortality but only improves the long-term functional outcome. On the other hand, by improving the functional status it can affect a better long-term prognosis for patients who survived hospitalization [18].

In Poland, which represents a middle-income country, in the years from 2006 to 2012 there was a decrease in SDR from AMI by 30 % and from stroke by just 18 % [1]. Recent data from Poland indicate a low in-hospital mortality due to AMI and still high (up to 15 %) post-AMI mortality in the first year of follow-up beyond the acute phase, which suggests a need to improve the quality of secondary prevention [13].

Our community-based study is the first published project comparing long-term survival of randomly selected IS and AMI subjects, for a sufficient length of time and in sufficiently large numbers for accurate statistics.

The results obtained in our study indicate a significantly higher post-IS mortality compared to AMI. This difference is largely due to lower survival among men after IS. It is worth noting that the results concerning long-term mortality rates are worse for post-IS men regardless of the method of their analysis (crude rates, SDRs, SMRs, survival probability in Kaplan–Meyer estimates, Cox model).

The analysis of SDRs in periods of time (Table 2) shows that the rates for the population of post-AMI and post-IS throughout the 10-year follow-up period are significantly higher than for the reference population. The values reach their peak in the first year from event. While after the first year of follow-up their value falls in the AMI group, for a similar decline in the IS group there is a delay of 2 years. Mortality rates in the studied groups of patients after IS and AMI exceed their respective annual SDR for the reference population, for men by seven times and for women ten times, and in the first year of follow-up as much as by twelve times (men) and fifteen times (women).

In general, our results are in agreement with those of previous investigations showing that the highest risk for death is in the first year from cardiovascular event. However, those investigations show the results separately for AMI and IS.

In the Oxfordshire Vascular Study (OVS) 675 patients with a first stroke were followed up for up to 6.5 years, and the relative risk of death was found to vary between 1.1 and 2.9 at 2–6 years after the stroke [9]. In the Perth Community Stroke Study, in which 362 patients with a first stroke were followed up for 5 years, the relative risk for death beyond 1 year after the stroke was between 2.0 and 2.3 [14]. In the Danish MONICA Study (DMS) the risk of death between 4 weeks and 12 months after the first stroke was 18.1 % (almost 2 times higher than in our study, but there were post-subarachnoid haemorrhage and post-intracranial haemorrhage subjects included in the Danish group). In DMS a SMR was ≥2.0 for as long as 5–15 years after the initial stroke [19]. In contrast to DMS, in our study there was an increase of risk of death for the period 8–10 years since event, probably due to population aging (but in both studies mean age was similar). Both in our study and in the Perth study, the highest risk of dying in the first year of follow-up, comparing to a reference population, accounted for the youngest patients, in Perth it was 200× higher for patients <45 years old, in our study 150×.

Cardiovascular diseases represented almost 2/3 of causes of post-stroke deaths in DMS and in our study. Similar deaths structure was found in the Perth and OVS studies. The proportion of deaths due to cancers was higher in our post-IS population than in DMS (22 vs. 13.5 %).

In the study of Smolina et al. the cohort of 371,619 30-day AMI survivors was followed during 7 years (median follow-up—2.8 years). The study showed that, as in the case of IS, survivors have a sustained worse prognosis than the general population [20]. Similar results come from Swedish and Canadian data (mortality rates about 3 times higher at 1 year after AMI in comparison to general population [21, 22]. Increased risk of death was particularly high in younger patients, which agrees with our study.

It is difficult to indicate a single cause for the higher mortality after IS when compared to AMI.

It is known that in the case of IS the functional state of the subjects and comorbidity are crucial for the long-term prognosis. The functional status is generally worse for IS, in comparison to AMI, due to frequent motor deficits and impaired cognitive function. These problems limit the quality of outpatient medical care. Another study conducted in Poland in 2006 comes in support of our thesis. It showed that post-IS subjects are much less likely to benefit from professional cardiac care, compared to patients after AMI [23]. This seems to be essential to explain the results obtained, because the most important role in reducing morbidity and long-term mortality is attributed to the proper control of the main cardiovascular risk factors, such as arterial hypertension, diabetes mellitus, hypercholesterolemia, smoking or—for IS—atrial fibrillation.

It should be noted, however, that potential differences in the quality of secondary prevention, (not identified in our study), do not justify a similar prognosis among post-IS women compared to post-AMI women. Similar conclusions were reached by the authors of MONICA, a multicentre study [15]. There is also a striking difference in terms of causes of death in the AMI and IS groups. While a significant proportion of CHD deaths in the IS group is not surprising and compatible with other publications, the distribution of causes of death in the AMI group in line with the population is striking. Perhaps after a given period of time from AMI, CHD ceases to be such a dominant health problem due to the increasingly effective prevention and coexistence of other diseases, especially cancer.

### Study limitations

It should be remembered that this study is only a description of a natural history of patients with IS and AMI and it does not explain the reasons for the difference in mortality and its causes. The study did not include data on medical treatment in the acute phase, pharmacological secondary prevention or prevalence of cardiovascular risk factors.

The advantages of the study are certainly strict criteria for selecting patients with correct diagnosis. The selection was based on the criteria of international scientific societies, as described in the methodology, not on the basis of the International Classification of Diseases (ICD) as has been the case in other works cited in our study.

The data loss of 5 % is a limitation of the study. It was a consequence of wrong or illegible information on patient data in hospital records (in years 2000–2005 there was no electronic database in hospitals in Gdynia).

This is one of the first ever studies using the representative method which enables a reliable epidemiological assessment at a relatively low cost, owing to a precise sampling (with the reservation that the results of the study do not reflect the global situation in Poland).

It would be valuable to conduct a similar study taking into account an accurate baseline evaluation of risk factors, the applied secondary prevention and re-occurrence of non-fatal cardiovascular events.
